# Survival of African Swine Fever Virus in Excretions from Pigs Experimentally Infected with the Georgia 2007/1 Isolate

**DOI:** 10.1111/tbed.12381

**Published:** 2015-06-24

**Authors:** K. Davies, L. C. Goatley, C. Guinat, C. L. Netherton, S. Gubbins, L. K. Dixon, A. L. Reis

**Affiliations:** ^1^ The Pirbright InstituteSurreyUK; ^2^ Department of Production and Population HealthThe Royal Veterinary CollegeHatfieldUK

**Keywords:** African swine fever virus, virus inactivation, virus survival, transmission

## Abstract

African swine fever virus (ASFV) causes a lethal haemorrhagic disease of swine which can be transmitted through direct contact with infected animals and their excretions or indirect contact with contaminated fomites. The shedding of ASFV by infected pigs and the stability of ASFV in the environment will determine the extent of environmental contamination. The recent outbreaks of ASF in Europe make it essential to develop disease transmission models in order to design effective control strategies to prevent further spread of ASF. In this study, we assessed the shedding and stability of ASFV in faeces, urine and oral fluid from pigs infected with the Georgia 2007/1 ASFV isolate. The half‐life of infectious ASFV in faeces was found to range from 0.65 days when stored at 4°C to 0.29 days when stored at 37°C, while in urine it was found to range from 2.19 days (4°C) to 0.41 days (37°C). Based on these half‐lives and the estimated dose required for infection, faeces and urine would be estimated to remain infectious for 8.48 and 15.33 days at 4°C and 3.71 and 2.88 days at 37°C, respectively. The half‐life of ASFV DNA was 8 to 9 days in faeces and 2 to 3 days in oral fluid at all temperatures. In urine, the half‐life of ASFV DNA was found to be 32.54 days at 4°C decreasing to 19.48 days at 37°C. These results indicate that ASFV in excretions may be an important route of ASFV transmission.

## Introduction

African swine fever virus (ASFV) is the cause of African swine fever (ASF) a frequently lethal, haemorrhagic disease of wild boar and domestic pigs. African swine fever is currently endemic in many African countries and Sardinia (OIE, 2014). African swine fever was recently introduced into the Trans Caucasus region, Russian Federation and several countries in eastern Europe. This followed the introduction of ASF, in 2007, to Georgia and subsequent spread into surrounding countries including Armenia, Azerbaijan and the Russian Federation (Beltrán‐Alcrudo et al., 2008; Sabirovic et al., 2008). Between 2012 and 2013, several outbreaks of ASF were reported in Ukraine and Belarus (OIE, 2012–2013). From January 2014 until present (April 2015), outbreaks of ASFV were detected in wild boar and domestic pigs in Lithuania, Poland and Latvia and wild boar in Estonia (OIE, 2014) marking the first outbreaks of ASFV in the continental European Union since it was eradicated in 1995 (Arias et al., 2002). In Sardinia, ASF has been endemic since its introduction in 1978 (Costard et al., 2009). These recent events make it essential to develop effective control strategies to prevent further spread of ASF into Europe. A key part of this will be to develop indirect and direct transmission models to facilitate the prediction of disease spread.

Transmission can occur through direct contact between sick and healthy animals or by contact with infectious excretions and secretions. Indirect transmission can also occur if healthy pigs ingest infected meat products or have contact with contaminated fomites (Mur et al., 2012). Indirect transmission is important in the spread of ASFV, for example the 2007 outbreak in Georgia was thought to have been caused by the improper disposal of infected pork from a ship at a Black Sea port (Beltrán‐Alcrudo et al., 2008).

In the current study, survival of ASFV in excretions from pigs was examined using samples obtained from experimental infection of pigs with the Georgia 2007/1 strain. This information will also help determine the potential diagnostic use of detecting ASFV DNA in excretions. In previous studies with other ASFV isolates, it was found that the inactivation of ASFV occurs more quickly in slurry than in medium (Turner and Williams, 1999) because no infectious virus was detected in slurry after 24 h at 4 or 22°C (Turner et al., 1999). However, studies carried out in slurry do not provide information on which excretions are likely to contain infectious virus and how long the virus can survive in them. Another set of experiments using different isolates found that faeces, urine and oral fluid collected from ASFV‐infected pigs can contain infective ASFV (Eustace Montgomery, 1921; Greig and Plowright, 1970). It has also been shown that ASFV in faeces can remain infective for at least eleven days when kept in the dark at room temperature (Eustace Montgomery, 1921). In a more recent study,^1^ it was found that the half‐life of ASFV in faeces was 1.7 days at 5°C and decreased to 0.2 days at 37°C. Faeces, urine and oral fluid can easily contaminate water sources, soil and animal pens. The assessment of the shedding and stability of ASFV in these excretions will be important in understanding the extent of environmental contamination that can occur. In the current study, we assessed the survival of ASFV DNA and infectious virus in faeces, urine and oral fluid from pigs infected with the Georgia 2007/1 isolate. Genome analysis of isolates obtained from the Russian Federation, Lithuania, Poland and Belarus indicated these were very closely related to the Georgia 2007/1 isolate (Malogolovkin et al., 2012; Gallardo et al., 2014). Thus, we considered the Georgia 2007/1 isolate representative of those isolates currently circulating in eastern Europe and the Russian Federation.

## Materials and Methods

### Infection of pigs with ASFV and sample collection

Eight Landrace cross‐domestic pigs were intramuscularly infected with ASFV isolate Georgia 2007/1 with 1 × 10^2^ 50% haemadsorbing doses (HAD_50_). An equal number of Landrace cross domestic pigs were placed in the same pen to act as contacts. This experiment was primarily carried out to assess the transmission of ASFV between infected and healthy animals (Guinat et al., 2014).

Faecal and urine samples were collected during the experiment, where possible, or during post‐mortem. Oral fluid was collected using cotton swabs which were incubated in 1 ml phosphate‐buffered saline (PBS) for 1 h. Fluid was collected after vortexing and the swab discarded. Oral fluid was also collected by pressing a rope, left in pens for pigs to chew on, through a mangle. Twenty‐six faecal and fifteen urine samples were collected between 0 and 11 days post‐infection (dpi). Twelve oral fluid samples, collected on cotton swabs, were obtained between 1 and 10 dpi. Samples were stored at −80°C before being tested by qPCR and virus titration for ASFV DNA and infectious virus.

### Processing of faeces

Faecal samples were separated into 500 mg aliquots and stored for a maximum of 98 days at 4 and 12°C or for a maximum of 70 days at room temperature (21–23°C) and 37°C. Samples were removed from storage at days 0, 1, 2, 3, 5, 7, 14, 21, 28, 35, 42, 70 or 98 depending on the availability of samples. Samples were diluted 1 : 10 with Earle's saline salt solution supplemented with 2% Field Antibiotics [1000 U/ml penicillin, 1 mg/ml streptomycin, 10 mg/ml kanamycin and 20 mg/ml amphotericin B (Sigma‐Aldrich, St Loius, MO, USA)] and 5% pig serum. Faecal samples were homogenized by vortexing with 1.4 mm ceramic beads (MP Biomedicals, Santa Ana, CA, USA) and centrifuged at 2370 ***g*** for 10 min to remove debris (Weesendorp et al., 2008). One millilitre of supernatant was removed and placed in a fresh tube before centrifugation at 18 000 ***g*** for 5 min to remove debris. The supernatants were individually analysed by virus titration and qPCR.

### Processing of urine and oral fluid

Urine and oral fluid samples were separated into 500 μl aliquots. Urine samples were stored for a maximum of 126 days at 4, 12, 21 or 37°C. Samples were removed from storage on days 0, 1, 2, 3, 5, 7, 14, 21, 28, 35, 42, 70, 98 or 126 depending on the availability of samples. Due to the small volume of oral fluid collected, all qPCR positive samples were mixed together. Oral fluid samples were stored for a maximum of 35 days at 4°C, 28 days at 12°C or 21 days at 21 and 37°C. Samples were removed from storage at days 0, 1, 2, 3, 5, 7, 14, 21, 28 or 35 depending on the availability of samples. Two per cent Field Antibiotic was added, and samples were centrifuged for 5 min at 18 000 ***g*** to remove debris. Supernatants were analysed for the presence of infectious ASFV and virus genome by virus titration and qPCR.

### Virus titrations

Virus titrations were carried out using pulmonary alveolar macrophages (PAMs) collected by lung lavage from uninfected pigs. Pulmonary alveolar macrophages were seeded at 1 × 10^5^ cells per well in a 96‐well plate. Fifty microlitres of sample supernatant was serially diluted seven times at a 1 : 10 dilution in Earle's saline salt solution. Hundred microlitres of each dilution was used to inoculate each of 3 wells of a 96‐well plate to obtain triplicate values. African swine fever virus Georgia 2007/1 was used as a positive control, and Earle's saline salt solution was used as a negative control. Plates were incubated at 37°C for 3 days.

Infected cells were identified by immunofluorescence using a monoclonal antibody against ASFV p30 protein. Medium was removed from each well, and cells were washed with PBS. Cells were fixed by immersing in 4% paraformaldehyde for 1 h at room temperature and then washed with PBS. Cells were permeabilized with 100 μl PBS + 0.2% Triton X‐100 per well for 5 min at room temperature and then washed with PBS. Cells were incubated with monoclonal antibody anti‐P30 (C18) diluted 1 : 3000 in PBS containing 1% bovine serum albumin (BSA) for 1 h at room temperature and then washed with PBS. Cells were incubated with anti‐mouse Alexa Fluor 488 diluted 1 : 500 in PBS containing 1% BSA for 1 h at room temperature and then washed with PBS. Plates were viewed under a fluorescent microscope, and the virus titre was determined using the Spearman–Kärber formula. The detection threshold for faeces was determined as 1 × 10^2.83^ TCID_50_/g (50% tissue culture infectious doses) and for urine and oral fluid was 1 × 10^1.83^ TCID_50_/ml. The difference in the thresholds is due to the fact that the faeces samples had to be diluted in medium before processing, while urine was used without dilution.

### DNA extraction and quantitative PCR (qPCR)

DNA was extracted from 100 μl of sample supernatant using the KingFisher Flex Extraction System (Thermo Scientific) using the MagVet^™^ Universal Isolation Kit (Life Technologies) following protocol NM_LSI_RRC96. Each extraction was carried out in duplicate and contained ASFV Georgia 2007/1 as a positive control and PBS as a negative control. Extracted DNA was stored at 4°C until it could be analysed by qPCR. qPCR was carried out on a Stratagene Mx3005P (Agilent Technologies, Santa Clara, CA, USA) following a protocol modified from King et al., 2003 as described by King et al., 2011. A standard curve was constructed by the serial dilution of control plasmid, and genome copies were calculated from the standard curve based on the cycle threshold (*C*_t_) values. *C*_t_ values >40 were considered negative.

### Statistical analysis

Significant differences between the average viral titre detected in blood samples collected from animals on days that they were positive or negative for infectious ASFV or ASFV genome in faecal, urine or oral fluid samples were tested with two‐way anova followed by Sidak's multiple comparisons tests using GraphPad Prism version 6.00 for Windows (GraphPad Software, La Jolla, CA, USA, www.graphpad.com).

The survival of viable ASFV assayed by virus titration was analysed using the R statistical software using packages ‘MASS’ and ‘survival’ (R‐Core‐Team). Log‐transformed virus titres were used to create a linear mixed model with sample as random effect and temperature as a continuous variable. The detection threshold was incorporated in the analysis by left‐censoring those observations in which the level of virus was below the detection threshold.

The survival of ASFV DNA assayed by qPCR was analysed using the R statistical software using packages ‘MASS’ and ‘nlme’ (R‐Core‐Team). Log‐transformed virus titres were used to create a linear mixed model fit by maximum likelihood, with sample as random effect and temperature as a continuous variable. Half‐life of viable ASFV and ASFV DNA was calculated from the models using the formula: Half‐life = −log(2)/slope. All samples that were positive for ASFV infectious virus were also positive for ASFV DNA.

## Results

### Detection of ASFV in excretions from infected pigs

As shown in Table 1, 2/26 faecal samples, 5/15 urine samples and 5/12 oral fluid samples collected, tested positive for ASFV DNA by qPCR. From these, 2/26 faecal samples and 3/15 urine samples were positive for infectious ASFV. However, no oral fluid samples were positive by virus titration for infectious ASFV. Faecal samples that were positive for infectious ASFV and ASFV DNA were detected from the first day of fever (>40°C). One urine sample was positive for ASFV DNA the day prior to the onset of fever, but infectious ASFV was only detected in urine from the first day of fever. In oral fluid, ASFV DNA was detected in samples from the start of fever.

**Table 1:** Number of samples collected of each sample type on days relative to the onset of pyrexia in infected pigs. The total number of samples collected of faeces, urine and oral fluid relative to the onset of fever in infected pigs is shown. These samples were tested by quantitative PCR (qPCR) and virus titration (VT) and the day that the samples tested positive for ASFV are also shown relative to the onset of pyrexia

	Sample type	Onset of Pyrexia (days)					Total
		−1	0	1	2	3	
Samples collected	Faeces	4	6	9	6	1	26
	Urine	2	4	5	4	–	15
	Oral fluid	–	3	5	4	–	12
qPCR positive	Faeces	–	1	–	1	–	2
	Urine	1	2	1	1	–	5
	Oral fluid	–	2	2	1	–	5
VT positive	Faeces	–	1	–	1	–	2
	Urine	–	2	–	1	–	3
	Oral fluid	–	–	–	–	–	–

To assess whether a higher viraemia was associated with a higher probability of collecting positive excretions, the average viral titre detected in blood samples (Guinat et al., 2014) collected from animals on days that they were either positive or negative for infectious ASFV or ASFV genome in faecal, urine or oral fluid samples was compared. The average virus titre in blood on the same day as faecal samples that were negative for ASFV DNA (*n* = 22) was 5.75 × 10^6^ and for those faecal samples that were positive for ASFV DNA (*n* = 2) was 4.96 × 10^6^. Average virus titres in blood were 8.88 × 10^6^ for urine samples (*n* = 10) that were negative for ASFV and 4.36 × 10^6^ for urine samples (*n* = 4) that were positive for ASFV. Differences in virus titres in blood for those faecal and urine samples that were positive for ASFV compared to those that were negative were not significant (*P *≥* *0.1).

### Survival of infectious ASFV in excretions

The survival of infectious ASFV in faeces and urine at different temperatures is shown in Figure S1. Infectious ASFV could be detected for up to 5 days at 4°C and 12°C, 3 days at 21°C and 1 day at 37°C in faeces (Figure S1). In urine, infectious ASFV could be detected for up to 5 days at 4°C, 12°C and 21°C and 1 day at 37°C (Figure S1). The half‐life of infectious ASFV in faeces and urine at different temperatures is shown in Table 2. The effect of temperature on the survival of ASFV in faeces and urine over time was statistically significant (*P* < 0.001).

**Table 2:** Half‐life of viable ASFV and ASFV DNA in excretions at different temperatures. Half‐life of viable ASFV and ASFV DNA in faeces, urine and oral fluid stored at 4, 12, 21 and 37°C

Half‐life of ASFV (days)										
Sample type	Virus titration				Number Samples Tested	qPCR				Number Samples Tested
	Temperature					Temperature				
	4°C	12°C	21°C	37°C		4°C	12°C	21°C	37°C	
Faeces	0.65	0.50	0.39	0.29	2	9.95	9.48	9.00	8.25	2
Urine	2.19	1.07	0.68	0.41	3	32.54	27.99	24.18	19.48	5
Oral fluid	–	–	–	–	–	2.75	2.72	2.67	2.60	5

Experimental infections with the moderately virulent Kenyan ASFV isolate Ken05/Tk1 showed that inoculation with 10 HAD_50_ (equivalent to TCID_50_) via the intramuscular route or oro‐nasal route resulted in ASF disease in all inoculated pigs. Similarly, immunization of pigs with 10 HAD_50_ of the Armenian ASFV isolate Arm07 via the intramuscular route resulted in all inoculated pigs developing ASF disease (Gallardo et al., 2013). The Arm07 isolate is closely related to the Georgia 2007/1 isolate used in this study. Therefore, 10 HAD50 was estimated to be the minimum infectious dose for the Georgia 2007/1 strain. The estimated duration of survival of viable ASFV above this infectious dose was calculated using the half‐life value and the mean initial viral titre. The mean initial titre of faecal samples was 1 × 10^4.83^ TCID_50_/g, and the mean initial titre of urine samples was 1 × 10^2.94^ TCID_50_/ml. The estimated duration of survival of infectious ASFV is shown in Table 3. With these initial titres, infectious ASFV can be estimated to survive in faeces from 8.5 days at 4°C to 3.7 days at 37°C and around 15.3 days at 4°C to 2.9 days at 37°C in urine.

**Table 3:** Estimated duration of survival of infectious ASFV in excretions at different temperatures. Estimated duration of survival of infectious ASFV in faeces, urine and oral fluid stored at 4, 12, 21 and 37°C, calculated assuming an infectious dose of 10 HAD_50_ initial mean viral titre of each sample type and half‐life value (Table 2) for each sample type at 4, 12, 21 and 37°C

Estimated survival of ASFV (days)					
Sample type	Viable ASFV				
	Mean initial titre (TCID_50_)	Temperature			
		4°C	12°C	21°C	37°C
Faeces	1 × 10^4.83^	8.5	6.5	5.1	3.7
Urine	1 × 10^2.94^	15.3	7.5	4.8	2.9
Oral Fluid	–	–	–	–	–

### Survival of ASFV DNA in excretions

The mean initial titre of ASFV in faeces was 1 × 10^6.52^ genome copies per gram. In urine, the mean initial titre of ASFV was 1 × 10^4.38^ genome copies per ml and for oral fluid the initial titre of ASFV was 1 × 10^4.72^ genome copies per ml. The survival of ASFV DNA in faeces, urine and oral fluid can be seen in Figure S2. In faeces, ASFV DNA could be detected up to at least day 98 at 4°C and 12°C and up to 35 days at 21°C and 37°C (Figure S2). In urine, ASFV DNA could be detected for at least 126 days at all temperatures (Figure S2). In oral fluid, ASFV DNA could be detected for 35 days at 4°C, 14 days at 12°C and 21°C. No ASFV DNA could be detected in oral fluid after storage at 37°C (Figure S2). The half‐life of ASFV DNA in faeces, urine and oral fluid can be seen in Table 3. The effect of temperature on the on the survival of ASFV DNA in urine faeces and oral fluid was statistically significant (*P* < 0.001).

## Discussion

This study assessed the shedding and survival of infectious ASFV and ASFV DNA in excretions collected from pigs infected with Georgia 2007/1 isolate. The shedding of ASFV in excretions begins at approximately the same time as the onset of fever. This was also observed in a previous study in which infected faeces were detected from the onset of fever (Greig and Plowright, 1970) or the onset of other clinical signs (de Carvalho Ferreira et al., 2012). Detection of ASFV DNA in blood is usually correlated with onset of clinical signs. To determine whether high viral titre in blood increased the chance of collecting excretion samples positive for ASFV genome or virus, the average viral titre detected in blood samples (Guinat et al., 2014) collected from animals on days that they were positive or negative for infectious ASFV or ASFV genome in excretions was compared. As no significant difference was obtained, this suggests that the level of viraemia has no effect on the production of infectious excretions. Possibly levels of replication in tissues in the local area may be an important factor.

Only one urine sample positive for ASFV was obtained outside of this period, the day before the start of fever. This animal had reached the humane end point the day after the positive sample was collected and was culled.

The half‐life for ASFV in urine was shown to be longer than the half‐life in faeces and oral fluid for both infectious virus and viral DNA, suggesting urine is the most stable medium tested for ASFV survival. Differences in survival times for infectious virus may result from the relative levels of enzymes, including proteases or lipases, which inactivate virus in the different samples. These enzymes may be derived from bacteria present. The half‐life for ASFV DNA in excretions is much longer than the half‐life for viable ASFV. Thus, the detection of ASFV DNA does not always predict the detection of infectious ASFV, but may confirm an infected population.

The collection of unpreserved field faecal samples as an alternative non‐invasive surveillance method of wild boar and free‐ranging pigs has been suggested as the survival of ASFV DNA in faeces can be long even at temperatures above room temperature^1^ (de Carvalho Ferreira et al., 2014). In a previous study, ASFV was detected in 53% of faecal samples from pigs infected with the highly virulent Brazil'78 ASFV isolate (de Carvalho Ferreira et al., 2014). However, in the present experiment, only 8.7% of the faeces collected from viraemic animals were positive. This suggests that the analysis of faecal samples would not be a sensitive method to use in the surveillance for strains of ASFV from eastern Europe, the Russian Federation or Trans Caucasus region in wild boar or free‐ranging pig populations. This observation suggests there may be differences in the pathogenesis resulting from infection with the Georgia 2007/1 isolate compared to the Brazil'78 isolate that may result in more frequent excretion of infectious virus in faeces from Brazil'78 isolate.

Half‐live values obtained in previous studies for survival of viable ASFV in faeces are similar to those observed in the present study. However, the half‐life value at lower temperatures was slightly longer, 1.7 days at 5°C in the previous study compared to 0.65 days at 4°C in the current study. The half‐life values for ASFV DNA in urine and faeces were also obtained previously and showed a greater stability of ASFV DNA in faeces and less stability of ASFV DNA in urine compared to observations in the present study.

Previously faecal samples collected from ASFV‐infected pigs were found to be infectious after storage at room temperature for 11 days (Eustace Montgomery, 1921). In the present study, faecal samples were estimated to remain infective for 5.1 days at room temperature. This difference may be due to the fact that the samples in the previous study were kept in the dark which may provide protection from damage caused to the virus by ultraviolet light.

The detection of viral DNA but no viable virus in oral fluid samples may have been due to the titre of infectious virus being lower than the detection threshold of the virus isolation test used. Viable virus was previously detected in saliva at 10^0.9^ HAD_50_/ml (Greig and Plowright, 1970), below the detection threshold for the method used in the present study. Viral DNA found on swabs may also have been due to residual contamination of the pig's mouth.

Only a relatively small proportion of samples collected contained infectious ASFV. However, as the infectious dose of ASFV via the oro‐nasal route was estimated to be 10 HAD_50_ (Gallardo et al., 2013), even a small amount of infective material could lead to transmission. Infectious material could be transported to other pens or farms on clothing, footwear, equipment and machinery. Faeces and urine can easily contaminate food and water sources for other animals in the same pen. It can therefore be concluded that the excretion of ASFV in the faeces and urine of infected pigs may be an important route of transmission of ASFV between domestic pigs.

## Supporting information


**Figure S1.** Survival of Infectious ASFV in faeces and urine stored at different temperatures. Graphs show the survival of infectious ASFV in faeces stored at different temperatures (Panel a, b, c, d), The red line represents the detection threshold at 1 × 10^2.83^ TCID_50_/g and the survival of infectious ASFV in urine (Panel e, f, g, h), with the black line representing the detection threshold at 1 × 10^1.83^ TCID_50_/ml.

**Figure S2.** Survival of ASFV DNA in excretions stored at different temperatures. Graphs show the survival of ASFV DNA in faeces (Panels a, b, c, d), the survival of ASFV DNA in urine (Panel e, f, g, h) and the survival of ASFV DNA in oral fluid (Panel i, j, k, l) stored at 4°C (Panels a, e, i), 12°C (Panels b, f, j), 21°C (Panels c, g, k) and 37°C (Panels d, h, l).

## Note

1 See Thesis Cardoso de Carvalho Ferreira, H. ‘Towards an improved understanding of African swine fever virus transmission’. (2013): http://dspace.library.uu.nl/handle/1874/282320 (accessed 30 March 2015).
