# Supplementary figures and images for: Survival of African Swine Fever Virus in Excretions from Pigs Experimentally Infected with the Georgia 2007/1 Isolate
Source: Transbound Emerg Dis. 2015 Jun 24;64(2):425–31. doi: 10.1111/tbed.12381 (PMC5347838; doi:10.1111/tbed.12381)

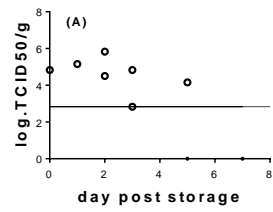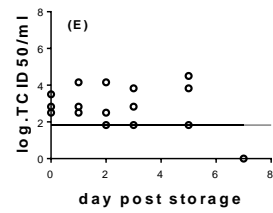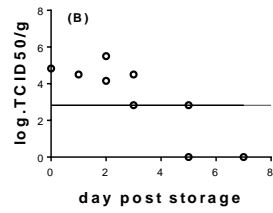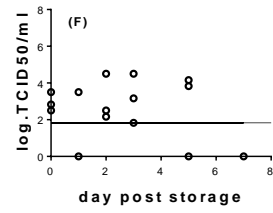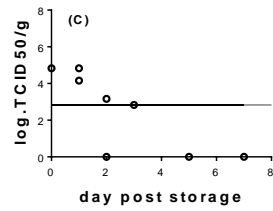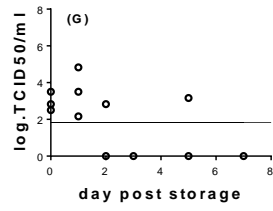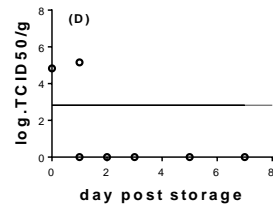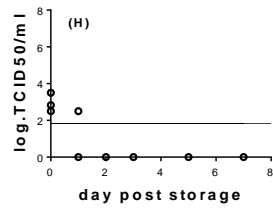

Supplement: Supplementary file 1 — Figure S1. Survival of Infectious ASFV in faeces and urine stored at different temperatures. Graphs show the survival of infectious ASFV in faeces stored at different temperatures (Panel a, b, c, d), The red line represents the detection threshold at 1 × 102.83 TCID50/g and the survival of infectious ASFV in urine (Panel e, f, g, h), with the black line representing the detection threshold at 1 × 101.83 TCID50/ml. [file TBED-64-425-s001.pdf]

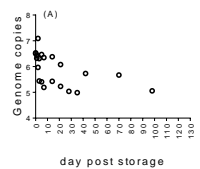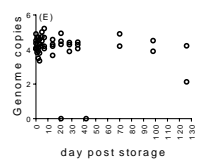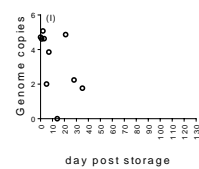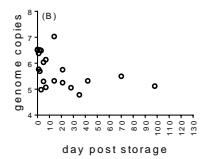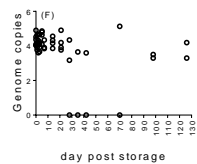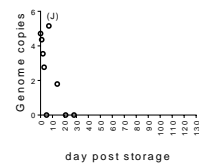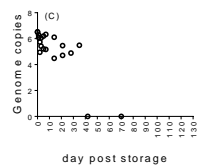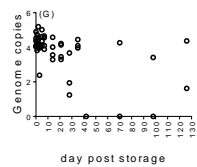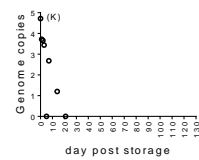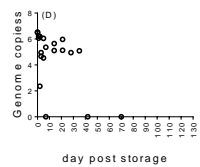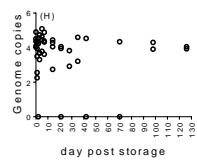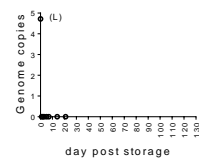

Supplement: Supplementary file 2 — Figure S2. Survival of ASFV DNA in excretions stored at different temperatures. Graphs show the survival of ASFV DNA in faeces (Panels a, b, c, d), the survival of ASFV DNA in urine (Panel e, f, g, h) and the survival of ASFV DNA in oral fluid (Panel i, j, k, l) stored at 4°C (Panels a, e, i), 12°C (Panels b, f, j), 21°C (Panels c, g, k) and 37°C (Panels d, h, l). [file TBED-64-425-s002.pdf]
